# The First Use of a ReX_5_ Synthon to Modulate Fe^III^ Spin Crossover via Supramolecular Halogen⋅⋅⋅Halogen Interactions

**DOI:** 10.1002/chem.202001668

**Published:** 2020-08-13

**Authors:** Rebecca Busch, Anthony B. Carter, Konstantis F. Konidaris, Irina A. Kühne, Ricardo González, Christopher E. Anson, Annie K. Powell

**Affiliations:** ^1^ Institute of Inorganic Chemistry Karlsruhe Institute of Technology Engesserstrasse 15 76131 Karlsruhe Germany; ^2^ School of Chemistry University of Southampton University Road Southampton SO17 1BJ UK; ^3^ Department of Pharmacy University of Patras 26504 Patras Greece; ^4^ School of Physics University College Dublin (UCD) Belfield Dublin 4 Ireland; ^5^ Cátedra de Química Inorgánica Departamento Estrella Campos Facultadde Química Universidad de la República Avda. General Flores 2124 CC 1157 Montevideo Uruguay; ^6^ Institute of Nanotechnology Karlsruhe Institute of Technology Hermann von Helmholtz Platz 1 76344 Eggenstein-Leopoldshafen Germany

**Keywords:** cooperative effects, halogen bonding, noncovalent interactions, self-assembly, spin crossover

## Abstract

We have added the {Re^IV^X_5_}^−^ (X=Br, Cl) synthon to a pocket‐based ligand to provide supramolecular design using halogen⋅⋅⋅halogen interactions within an Fe^III^ system that has the potential to undergo spin crossover (SCO). By removing the solvent from the crystal lattice, we “switch on” halogen⋅⋅⋅halogen interactions between neighboring molecules, providing a supramolecular cooperative pathway for SCO. Furthermore, changes to the halogen‐based interaction allow us to modify the temperature and nature of the SCO event.

## Graphical abstract

**Inorganic synthon controls SCO behavior**: Incorporating an {Re^IV^X_5_}^−^ synthon into an Fe^III^ system provides a highly directional supramolecular design using halogen⋅⋅⋅halogen interactions. Removal of solvent from the crystal lattice “switches on” these interactions, providing a long‐range cooperative pathway ideal for an SCO event.

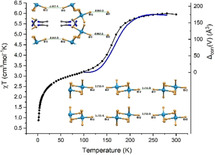

## Introduction

Supramolecular chemistry is a rapidly expanding area of research,^1^ having seen the award of two Nobel prizes within the last 30 years.^2^ Whilst this means the phenomenon is well known, the important role it plays within inorganic structural chemistry is only beginning to be fully appreciated. The most common supramolecular interaction utilized is hydrogen bonding, which is fundamental for all life by allowing liquid water to exist over a wide range of temperatures and pressures.^3^ In addition to hydrogen bonding, there are other intermolecular interactions such as π–π,^4^ van der Waals,^5^ and the less common halogen,^6^ chalcogen,^7^ tetrel,^8^ or pnictogen bonds.^9^ Spin crossover (SCO) complexes are a class of materials which exhibit molecular bistability where both the high spin (HS) and low spin (LS) electronic configurations of certain d^4^–d^7^ transition metal complexes can be accessed in response to external stimuli such as light, temperature, or pressure.^10^ In solid systems the nature of the spin‐transition is steered by the local coordination sphere as well as the long‐range order (supramolecular bonds).^11^ The latter can be removed by isolating the molecules, either through crystal engineering approaching or dissolution.^12^ The role that supramolecular interactions play in SCO complexes is well established, with both the strength and direction of these leading to extensive changes,^13^ in particular solvent mediated interactions have been recognized as important.^14^ It has recently been recognized that halogen bonds are a significant noncovalent bonding pathway for transmitting or supporting information transfer, whilst also providing stability to supramolecular systems.^15^ The high directionality associated with halogen bonds should prove a useful tool in SCO research, allowing systems to be designed with a high degree of control over the direction of information propagation. In recent years studies by Fourmigué have pointed towards this direction, demonstrating the potential for halogen‐based supramolecular interactions within SCO research.^16^

**Scheme 1:**
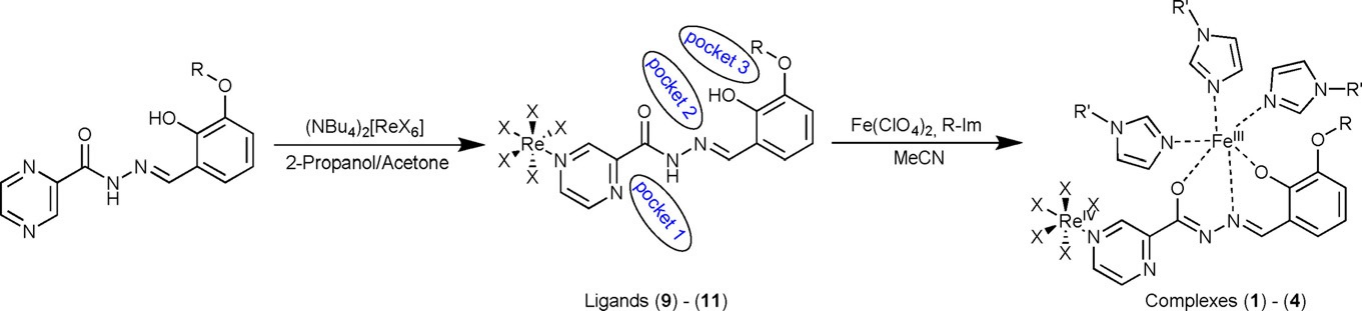
Synthetic route for the preparation of ligands (**9**)–(**11**), [Re*X*_5_(*R*pch)]^−^ (containing the a {ReX_5_}^−^ synthon), and the complexes (**1**)–(**4**).

**Figure 1:**
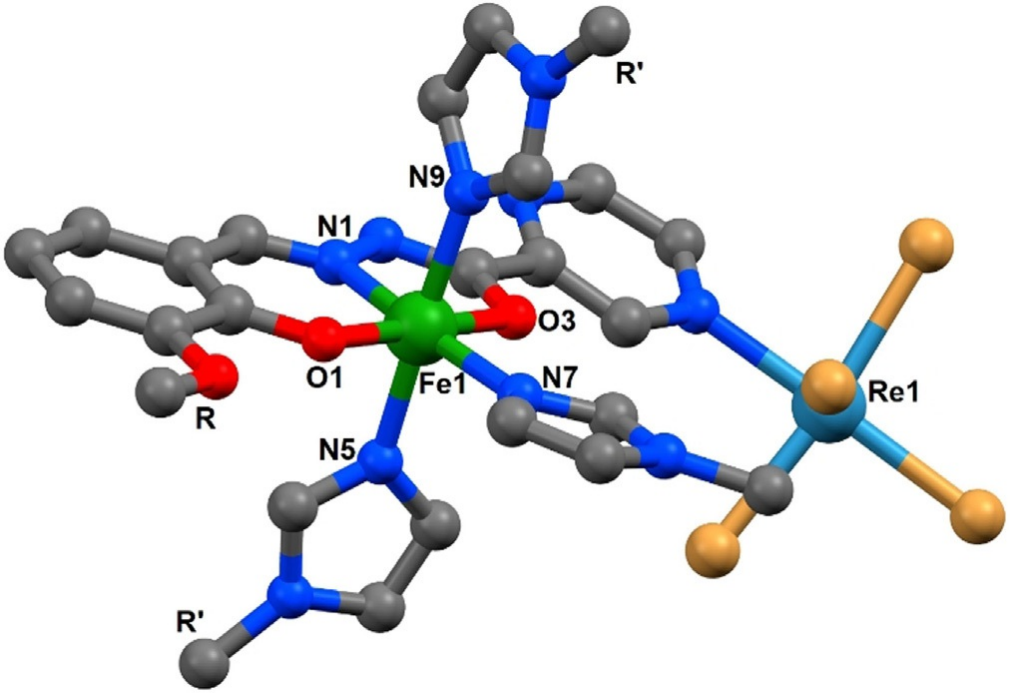
The molecular structure in the desolvated compound (**1**)d_100.

In an attempt to produce a bimetallic 3d–5d molecule using a {ReX_5_}^−^ synthon and a Schiff‐base contacting ligand with flexible binding pockets, similar to those previously used by our group^17^ (Scheme 1), we produced a family of new heterometallic Fe^III^‐5d systems. In practice we found that the transition metal preferentially occupies pocket 2, giving a *mer*‐coordinated system with the general formula [Re^IV^ *X*_5_(μ‐*R*pch)Fe(*R’*‐Im)_3_] where H_2_ *R*pch has the formula (*E*)‐*N*’‐(2‐hydroxy‐3‐*R*‐benzylidene)pyrazine‐2‐carbohydrazide, and *R’*‐Im has the formula 1‐*R’*‐imidazole (Figure 1, Table 1).

Whilst the use of the {ReX_5_}^−^ synthon has been seldom reported^18^ we noted that by removing competing intermolecular interactions such as hydrogen bonds we promote halogen⋅⋅⋅ halogen interactions, allowing us to switch on supramolecular interactions which enable SCO events. A careful multipronged investigation involving modifications to the halogen, the ligand and co‐ligand, as well as the synthesis of the analogous Co^III^ compounds (**5**)‐(**8**) (see ESI), showed that the SCO properties of the Fe^III^ center could be readily modified. Crystal structures are labelled with the general name “(**Compound**)w/d_TEMP”, where w or d denotes “wet” (solvated) or “dry” (desolvated) crystals, and TEMP is the temperature of the structural determination.

## Results and Discussion

**Figure 2:**
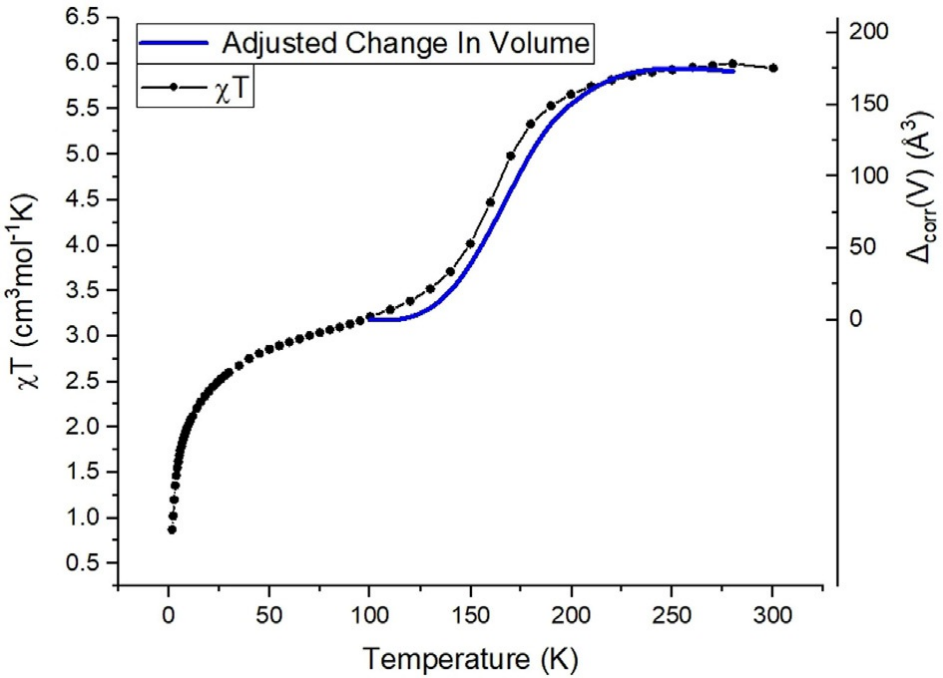
*χT* plot with overlaid adjusted unit cell volume (see ESI) for complex (**1**) showing the close correspondence of the values. Magnetic data collected under an applied DC field of 0.1 T.

Single crystal X‐ray analysis of complex (**1**)w_100 shows that the complex crystallizes in the space group *C*2/*c* with *Z*=8. Complex (**1**) was chosen as a representative of the series with structural details of complexes (**1**)–(**4**) including halogen bond parameters in the ESI. The asymmetric unit contains a [Re*Br*_5_(μ‐*Me*Opch)Fe(*Me*‐Im)_3_] complex with the {Re^IV^Br_5_} synthon bound to a nitrogen atom of the pyrazine ring of the deprotonated MeOpch ligand. The central Fe^III^ ion is coordinated to the ligand through an azine nitrogen (N1), a carbonyl oxygen (O3), and a hydroxy oxygen (O1), whilst three additional Me‐Im co‐ligands complete the coordination sphere through nitrogen atoms N5, N9, and N7. In addition to this there were two interstitial MeCN molecules within the lattice connected through nonclassical hydrogen bonds. The shortest intermolecular Br⋅⋅⋅Br distance was 4.663 Å and is therefore too long to be considered a halogen bond, and is unlikely to contribute to information exchange.^19^ The N_4_O_2_ coordination sphere of the Fe^III^ ion is often observed in SCO active compounds,^20^ so this complex warranted further investigation and a structure was collected at 280 K, (**1**)w_280. Fe^III^−N bond lengths are characteristic of the spin state (LS≈1.95 Å and HS≈2.15 Å)^21^ and crystallographic analysis of the complex showed that the system was in the HS state at both temperatures, with the average Fe−N bonds being 2.098 Å and 2.114 Å at 100 K and 230 K, respectively. However, magnetic analysis of complex (**1**) using a SQUID magnetometer revealed an SCO active system with an incomplete transition occurring between 220 K and 90 K, from fully HS to only 22 % of molecules in the HS electronic configuration with T_1/2_=155 K (Figure 2). This initial result was surprising given that single crystal X‐ray diffraction data had indicated a HS state at 100 K. The sample was removed from the SQUID and new single crystal X‐ray diffraction data were collected. The sample (**1**)d_100 crystallized in the same space group, *C*2/*c*, with the same neutral metal complex in the asymmetric unit. However, the average Fe−N bond length (1.982 Å) indicated the complex was now at least partially in the LS state; it was also observed that the sample had desolvated (between measurements) with loss of the interstitial MeCN molecules (confirmed by elemental analysis) whilst retaining crystallinity. Crystallographic data were also collected at 140, 180, 230 and 280 K and by plotting the adjusted change in unit cell volume (see ESI for details) against the temperature the SCO event clearly correlates with the changes in the crystal structure (Figure 2).

**Figure 3:**
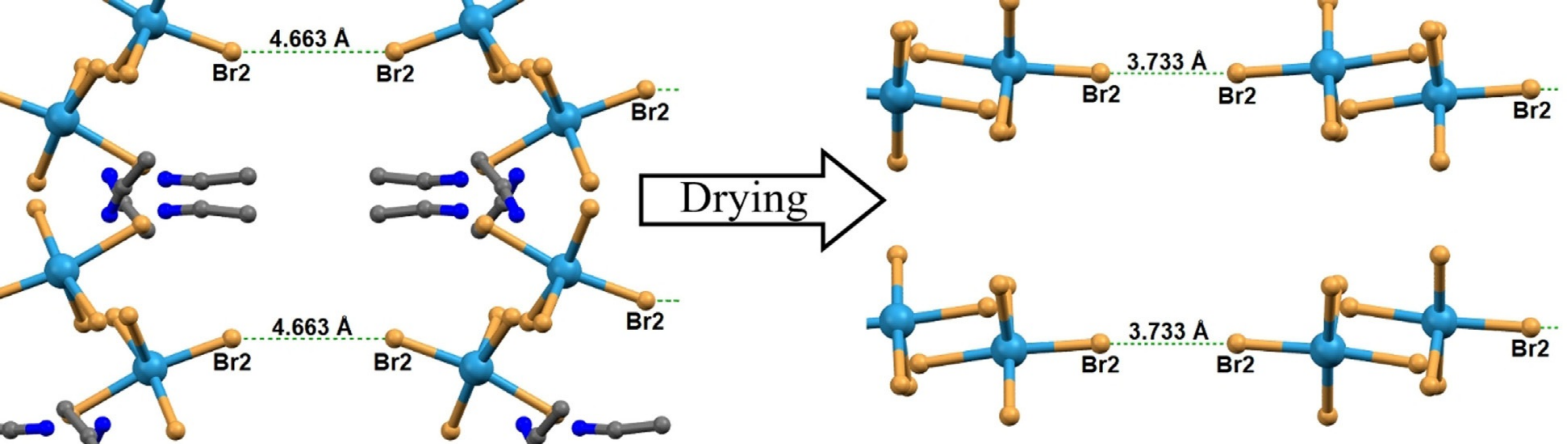
Packing rearrangement of (**1**) before (left) and after (right) loss of solvent molecules, both crystal structures measured at 100 K.

A more detailed investigation revealed that, by removing the lattice solvent and therefore the hydrogen bonding, halogen⋅⋅⋅halogen interactions become the dominant supramolecular effect (Figure 3). In the original solvated sample, the longer Br⋅⋅⋅Br distances and Re‐Br⋅⋅⋅Br angle blocks the formation of a bond (Br2⋅⋅⋅Br2’=4.663 Å, ∠Re‐Br⋅⋅⋅Br=160°, R_XB_=1.217), however the loss of the solvent leads to reorientation of molecules in the crystal resulting in a significant shortening of the Br⋅⋅⋅Br distances, “switching on” a type I halogen bond (Br2⋅⋅⋅Br2’=3.733 Å, ∠Re‐Br⋅⋅⋅Br=176°, R_XB_=1.009).^22^

**Figure 4:**
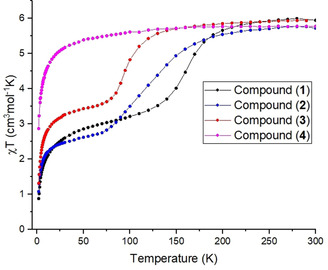
*χT* plots from the iron containing complexes (**1**)—(**4**) Data collected under an applied DC field of 0.1 T.

Complexes (**2**)–(**4**) were then synthesized to characterize the importance of these halogen⋅⋅⋅halogen interactions. The modifications are summarized in Table 1 and Figure 1 and were designed to provide additional insight. The replacement of a bromide for a chloride resulted in complex (**2**). Complex (**2**)w_100 also crystallized in the space group *C*2/*c* with the neutral metal complex now [Re*Cl*_5_(μ‐*Me*Opch)Fe(*Me*‐Im)_3_] together with two MeCN molecules in the asymmetric unit. Analysis of the Fe−N bond lengths (average: 2.111 Å) shows the iron(III) center to be in the HS state at 100 K. In this solvated sample there is again no appreciable halogen⋅⋅⋅halogen interaction, with the shortest Cl⋅⋅⋅Cl distance at 4.539 Å. Despite the apparent HS structure, magnetic investigation revealed that at 100 K the Fe^III^ is mostly LS (74 % LS) (Figure 4). Elemental analysis confirmed the crystal had lost the interstitial MeCN molecules indicating a similar effect to that seen in complex (**1**) with the halogen bond “switching on” upon loss of solvent. Since this sample lost crystal quality during desolvation, the exact nature of the halogen⋅⋅⋅halogen interaction could not be clearly defined. Previous work has ordered the strength of halogen bonds as F < Cl <Br < I.^23^ It is also well established that a decrease in cooperativity between metal centers results in less abrupt spin transitions.^24^ By exchanging the bromide ion with chloride, a weaker halogen bond has been induced; it is therefore expected that a transition would be more gradual. We can quantify the gradual nature of this transition when comparing it with complex (**1**). Both events start at 230 K from a *χT* value of ≈5.8 cm^3^ mol^−1^ K, however the T_1/2_ value is 33 K lower (T_1/2_=124 K) for (**2**) consistent with the gradual nature of the crossover which occurs over a wider temperature range (130 K and 170 K for (**1**) and (**2**), respectively).

**Table 1:** The formula of the reported compounds (**1**)–(**4**) colored for clarity.^[a]^

Compound	X=	R=	R’=
(**1**)	Br	MeO	Me
(**2**)	Cl	MeO	Me
(**3**)	Br	EtO	Me
(**4**)	Br	MeO	Et

[a] Note: Compounds (**5**)–(**8**) are the Co^III^ analogues of (**1**)–(**4**), respectively.

Complex (**3**) was produced by changing the non‐coordinated MeO group on the primary‐ligand for the sterically larger EtO group. (**3**)w_180 crystallized with a similar unit cell and packing, but in *P*2_1_/*n* due to loss of *C*‐centering. The asymmetric unit thus contains two [Re*Br*_5_(μ‐*Et*Opch)Fe(*Me*‐Im)_3_] complexes and a single molecule of MeCN. Both crystallographically independent complexes appear to be HS at 180 K with the average Fe‐N lengths of 2.116 Å and 2.101 Å for Fe1 and Fe2, respectively. In the complex containing Fe2 there is evidence of a weak type II halogen bond (Br7⋅⋅⋅Br10’ = 4.099 Å, ∠Re‐Br⋅⋅⋅Br = 131 & 119°, R_XB_ = 1.108), which is missing for the molecule containing Fe1, thus only half the molecules in the crystal lattice can utilize a halogen bond mediated process. Upon complete solvent loss, sample (**3**)d_180 undergoes a transformation to a structure with only one [Re*Br*_5_(μ‐*Et*Opch)Fe(*Me*‐Im)_3_] molecule in the asymmetric unit. In this case the halogen⋅⋅⋅halogen interaction is stronger than previously observed (Br5⋅⋅⋅Br5’=3.888 Å, ∠Re‐Br⋅⋅⋅Br=121°, R_XB_=1.051) involving all molecules in the crystal. SCO events are highly sensitive and this slight change in peripheral substituent from MeO to EtO is enough to modify the SCO properties.^25^ The magnetic data for the desolvated compound demonstrates a more abrupt incomplete SCO with 61 % of the metal centers reaching the LS occurring between 140 K to 60 K (80 K range) with a T_1/2_=98 K (Figure 4). The dramatic change in T_1/2_ highlights the suitability of this system to tune the SCO event towards a desired temperature.

**Figure 5:**
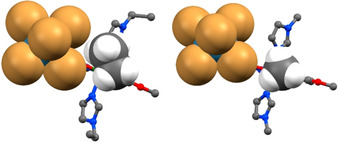
Representation of (**4**)d_180 (left) and (**1**)d_100 (right) shown in ball and stick with the “{ReX_5_}” and “imidazole *R’* group” in space filling mode demonstrating the steric hindrance induced by the ethyl group.

Finally, complex (**4**) which replaces the methyl group on the imidazole‐based secondary ligand, *R’*, with a larger ethyl group crystallizes without interstitial solvent, (**4**)d_180. In the case of (**3**) only one additional CH_2_ unit was added to the molecule in a position placing it at a maximum distance from the {ReX_5_}^−^ synthon. However, in complex (**4**) a total of three additional CH_2_ units were introduced pushing the molecules further apart. In addition, a methyl group on one imidazole points directly into the {ReX_5_}^−^ synthon (Figure 5) hindering the formation of a halogen bond (Br1⋅⋅⋅Br1’=4.455 Å, ∠Re‐Br⋅⋅⋅Br=167°, R_XB_=1.204). This results in a desolvated complex lacking halogen⋅⋅⋅halogen interactions and therefore without the desired SCO behavior (Figure 4).

The results for the desolvated complex (**4**) rule out the possibility that removing solvent alone is enough to induce SCO properties and provide further evidence that in these systems a halogen⋅⋅⋅halogen interaction is required. This highlights the key role of halogen bond mediated information exchange and demonstrates the potential for halogen bonding within SCO research.

As a classic halogen bond is defined as an attractive dipolar interaction between nucleophilic and electrophilic halogen centers, the symmetrical ReBr_5_⋅⋅⋅ReBr_5_ interactions we report here will clearly not be as strong, which is why we describe them as interactions rather than bonds. However, examination of the intermolecular geometries suggests that they can be validly described as weak halogen interactions. This is shown when they are “switched on” by solvent loss through a decrease in the Br⋅⋅⋅Br distances to what amounts approximately to the sum of their Van der Waals radii and the fact that the Re‐Br‐Br angles switch from 110–140° to values close to linear. These concomitant changes provide good evidence that for the relevance of these halogen⋅⋅⋅halogen interactions in these systems. Incorporating additional halogen interaction/bonding motifs offers a fruitful avenue of research into providing increased cooperativity between the Fe^III^ centers.

## Conclusions

Our systematic approach which utilizes magnetic measurements and detailed structural investigations to observe compare and contrast the properties of similar systems showing the presence or absence of halogen⋅⋅⋅halogen interactions leads to a clear and concise understanding of the role which non‐typical supramolecular interactions can play in magnetic switching. The use of the {ReX_5_}^−^ synthon in conjunction with a pocket ligand enabled us to provide an ideal Fe^III^ SCO environment coupled with the possibility to switch on halogen⋅⋅⋅ halogen interactions through desolvation of the crystals. This engendered long‐range noncovalent interactions. The successful implementation of this approach to providing a facile way to include highly directional supramolecular interactions paves the way for future research into the role of such interactions within SCO and magnetically interesting compounds.

## Experimental Section

The experimental section below covers the ligands (**9**) to (**11**) and compounds (**1**) to (**4**), with the Co^III^ analogues, compounds (**5**) to (**8**), presented in the ESI along with the full details of the analytical equipment.

### Synthesis of the (NBu_4_)[Re^IV^Br_5_(H_2_MeOpch)] ligand (9)

(NBu_4_)_2_ReBr_6_ (1.61 g, 1.40 mmol) and H_2_MeOpch (2.29 g, 8.40 mmol) were dissolved in a mixture of 2‐propanol/acetone (2:1, v/v, 150 mL) and heated to 75 °C for 2 hours. After cooling to room temperature, the solvent volume was reduced to ≈80 mL and the orange precipitate collected via filtration and dried in vacuo. Yield: 1.34 g (87 %). C_29_H_48_Br_5_N_5_O_3_Re (1098.44 g mol^−1^): Calculated C: 31.7 %, H: 4.4 %, N: 6.4 %, Found: C: 31.0 %, H: 4.3 %, N: 6.1 %. IR ($\tilde{\nu }$ cm^−1^): 2960 (m), 2932 (w), 2872 (w), 1683 (s), 1608 (w), 1528 (m), 1462 (s), 1406 (m), 1377 (m), 1353 (m), 1244 (vs), 1148 (s), 1077 (s), 968 (m), 952 (m), 905 (m), 881 (m), 781 (m), 629 (w), 482 (m). UV/Vis: (MeCN) (*λ* nm)=201, 220, 305, 339, 357 (sh), 590.

### Synthesis of the (NBu_4_)[Re^IV^Cl_5_(H_2_MeOpch)] ligand (10)

(NBu_4_)_2_ReCl_6_ (0.088 g, 0.10 mmol) and H_2_MeOpch (0.163 mg, 0.6 mmol) were dissolved in a mixture of 2‐propanol/acetone (2:1, v/v, 30 mL) and heated to 75 °C for 6 hours. After cooling to room temperature, the solution was filtered and left to stand under an inert atmosphere. After six days orange needles were collected via filtration and dried in vacuo. Yield: 0.067 g (67 %). C_29_H_48_Cl_5_N_5_O_3_Re (997.35 g mol^−1^): Calculated C: 42.2 %, H: 6.4 %, N: 7.0 %, Found: C: 40.9 %, H: 6.4 %, N: 7.0 %. IR ($\tilde{\nu }$ cm^−1^): 3490 (vw), 2960 (s), 2932 (m), 2873 (m), 1682 (vs), 1608 (m), 1576 (w), 1533 (s), 1464 (vs), 1407 (m), 1377 (m), 1354 (s), 1282 (s), 1246 (vs), 1147 (vs), 1076 (s), 1024 (m), 952 (s), 906 (m), 881 (m), 835 (w), 817 (w), 783 (m), 739 (vs), 486 (m), 344 (s). UV/Vis: (MeCN) (*λ* nm)=200, 221, 292, 353 (sh).

### Synthesis of the (NBu_4_)[Re^IV^Br_5_(H_2_EtOpch)] ligand (11)

(NBu_4_)_2_ReBr_6_ (0.080 g, 0.07 mmol) and H_2_EtOpch (0.120 g, 0.42 mmol) were dissolved in a mixture of 2‐propanol/acetone (2:1, v/v, 30 mL) and heated to 70 °C for 3 hours. After cooling to room temperature, the solvent volume was reduced to ≈10 mL and the orange precipitate collected via filtration and dried in vacuo. Yield: 0.064 g (82 %). C_30_H_49_Br_5_N_5_O_3_Re (1113.48 g mol^−1^): Calculated C: 32.4 %, H: 4.4 %, N: 6.3 %, Found: C: 32.6 %, H: 4.5 %, N: 6.3 %. IR ($\tilde{\nu }$ cm^−1^): 3298 (vw), 2953 (w), 2928 (w), 2868 (w), 1708 (s), 1607 (m), 1513 (s), 1461 (s), 1403 (s), 1377 (m), 1354 (m), 1255 (vs), 1153 (vs), 1111 (m), 1057 (s), 1023 (s), 948 (m), 922 (w), 882 (s), 778 (m), 732 (vs), 581 (s), 486 (s), 443 (w). UV/Vis: (MeCN) (*λ* nm)=221, 265, 305, 339, 357 (sh).

### Synthesis of [ReBr_5_(μ‐MeOpch)Fe(Me‐Im)_3_] (1)

A solution of Fe(ClO_4_)_2_⋅6 H_2_0 (7 mg, 0.02 mmol) in MeCN (5 mL) was slowly added to a solution of (NBu_4_)[ReBr_5_(H_2_MeOpch)] (**9**) (12 mg, 0.01 mmol) and 1‐Me‐Im (4 mg, 0.04 mmol) in MeCN (5 mL). The reddish‐brown clear solution was sealed and left to stand for three weeks until dark brown needle crystals were formed. Yield: 5 mg (48 %). C_25_H_28_Br_5_FeN_10_O_3_Re (1158.2 g mol^−1^): Calculated C: 25.9 %, H: 2.4 %, N: 12.1 %, Found: C: 26.5 %, H: 2.7 %, N: 12.7 %. IR ($\tilde{\nu }$ cm^−1^): 3123 (w), 1589 (m), 1536 (m), 1515 (m), 1466 (w), 1431 (s), 1349 (s), 1287 (m), 1252 (s), 1231 (m),1213 (m), 1156 (m), 1087 (vs), 1020 (w), 969 (w), 948 (m), 923 (w), 825 (w), 785 (w), 750 (vs), 655 (s), 618 (m), 584 (s), 509 (w), 416 (s), 370 (m). UV/Vis: (MeCN) (*λ* nm)=216, 281, 337, 350, 360.

### Synthesis of [ReCl_5_(μ‐MeOpch)Fe(Me‐Im)_3_] (2)

A solution of Fe(ClO_4_)_2_⋅6 H_2_0 (7 mg, 0.02 mmol) in MeCN (5 mL) was slowly added to a solution of (NBu_4_)[ReCl_5_(H_2_MeOpch)] (**10**) (12 mg, 0.01 mmol) and 1‐Me‐Im (3 mg, 0.03 mmol) in MeCN (5 mL). The reddish‐brown clear solution was sealed and left to stand for eight days until dark brown needle crystals were formed. Yield: 6 mg (54 %). C_25_H_28_Cl_5_FeN_10_O_3_Re (1158.2 g mol^−1^): Calculated C: 32.1 %, H: 3.0 %, N: 15.0 %, Found: C: 31.2 %, H: 3.0 %, N: 14.7 %. IR ($\tilde{\nu }$ cm^−1^): 2959 (vw), 2932 (vw), 2834 (vw), 1599 (s), 1548(s), 1509 (m), 1436 (s), 1354 (s), 1298 (m), 1242 (s), 1219 (vs), 1157 (s), 1081 (m), 1020 (w), 971 (w), 921 (w), 856 (m), 741 (vs), 629 (w), 552 (m), 492 (w), 442 (m), 404 (m), 360 (w). UV/Vis: (MeCN) (*λ* nm)=205, 282, 326 (sh), 338, 381, 477.

### Synthesis of [ReBr_5_(μ‐EtOpch)Fe(Me‐Im)_3_] (3)

A solution of Fe(ClO_4_)_2_⋅6 H_2_0 (35 mg, 0.12 mmol) in MeCN (10 mL) was slowly added to a solution of (NBu_4_)[ReBr_5_(H_2_EtOpch)] (**11**) (60 mg, 0.06 mmol) and 1‐Me‐Im (20 mg, 0.24 mmol) in MeCN (10 mL). The reddish‐brown clear solution was sealed and left to stand for four days until dark brown block crystals were formed. Yield: 5 mg (48 %). C_26_H_31_Br_5_FeN_10_O_3_Re (1172.67 g mol^−1^): Calculated C: 26.6 %, H: 2.6 %, N: 11.9 %, Found: C: 26.1 %, H: 2.4 %, N: 12.1 %. IR ($\tilde{\nu }$ cm^−1^): 3117 (w), 1606 (w), 1588 (m), 1532 (w), 1433 (m), 1391 (m), 1350 (m), 1280 (w), 1254 (s), 1211 (m), 1157 (m), 1086 (vs), 1023 (m), 946 (m), 901 (w), 844 (m), 783 (w), 741 (vs), 655 (s), 616 (m), 569 (m), 510 (m), 463 (w), 432 (m), 365 (w). UV/Vis: (MeCN) (*λ* nm)=195, 208, 221, 307, 335, 355, 388 (sh).

### Synthesis of [ReBr_5_(μ‐MeOpch)Fe(Et‐Im)_3_] (4)

A solution of Fe(ClO_4_)_2_⋅6 H_2_0 (22 mg, 0.06 mmol) in MeCN (10 mL) was slowly added to a solution of (NBu_4_)[ReBr_5_(H_2_MeOpch)] (**9**) (60 mg, 0.06 mmol) and 1‐Et‐Im (12 mg, 0.12 mmol) in MeCN (15 mL). The reddish‐brown clear solution was sealed and left to stand for one day until dark brown block crystals were formed. Yield: 31 mg (43 %). C_28_H_34_Br_5_CoN_10_O_3_Re (1153.26 g mol^−1^): Calculated C: 26.9 %, H: 2.6 %, N: 11.3 %, Found: C: 26.6 %, H: 2.7 %, N: 11.3 %. IR ($\tilde{\nu }$ cm^−1^): 3118 (w), 2976 (vw), 934 (vw), 1589 (m), 1550 (m), 1531 (m), 1462 (m), 1426 (m), 1350 (s), 1278 (m), 1245 (m), 1230 (m), 1215 (m), 1182 (w), 1158 (m), 1087 (vs), 1020 (m), 958 (m), 945 (m), 838 (m), 798 (w), 744 (vs), 660 (s), 590 (m), 567 (m), 512 (m), 431 (m), 359 (s). UV/Vis: (MeCN) (*λ* nm)=191, 209, 274 (sh), 338, 363, 392.

Deposition Numbers 1978010, 1978011, 1978012, 1978013, 1978014, 1978015, 1978016, 1978017, 1978018, 1978019, 1978020, 1978021, 1978022, 1978023, 1978024, 1978025, 1978026, 1978027, 1978028, and 1978029 contain the supplementary crystallographic data for this paper. These data are provided free of charge by the joint Cambridge Crystallographic Data Centre and Fachinformationszentrum Karlsruhe Access Structures service www.ccdc.cam.ac.uk/structures.

## Conflict of interest

The authors declare no conflict of interest.

## Supporting information

As a service to our authors and readers, this journal provides supporting information supplied by the authors. Such materials are peer reviewed and may be re‐organized for online delivery, but are not copy‐edited or typeset. Technical support issues arising from supporting information (other than missing files) should be addressed to the authors.

Supplementary
