# Supplementary material for: The First Use of a ReX5 Synthon to Modulate FeIII Spin Crossover via Supramolecular Halogen⋅⋅⋅Halogen Interactions
Source: Chemistry. 2020 Aug 13;26(51):11835–40. doi: 10.1002/chem.202001668 (PMC7540276; doi:10.1002/chem.202001668)
Supplement: Supplementary file 1 — Supplementary [file CHEM-26-11835-s001.pdf]

Chemistry–A European Journal

Supporting Information

The First Use of a ReX_5 Synthon to Modulate Fe^{III} Spin Crossover via Supramolecular Halogen···Halogen Interactions

Rebecca Busch+,[a] Anthony B. Carter+,[a, b] Konstantis F. Konidaris,[a, c] Irina A. Kühne,[a, d] Ricardo González,*[e] Christopher E. Anson,[a] and Annie K. Powell*[a, f]

Contents

Contents 1
1. Experimental Details 2
General Remarks 2
Synthesis of the $(NBu_4)[Re^{IV}Br_5(H_2MeOpch)]$ ligand (9) 2
Synthesis of the $(NBu_4)[Re^{IV}Cl_5(H_2MeOpch)]$ ligand (10) 2
Synthesis of the $(NBu_4)[Re^{IV}Br_5(H_2EtOpch)]$ ligand (11) 2
Synthesis of $[ReBr_5(\mu\text{-MeOpch})Fe(Me\text{-Im})_3]$ (1) 2
Synthesis of $[ReCl_5(\mu\text{-MeOpch})Fe(Me\text{-Im})_3]$ (2) 3
Synthesis of $[ReBr_5(\mu\text{-EtOpch})Fe(Me\text{-Im})_3]$ (3) 3
Synthesis of $[ReBr_5(\mu\text{-MeOpch})Fe(Et\text{-Im})_3]$ (4) 3
Synthesis of $[ReBr_5(\mu\text{-MeOpch})Co(Me\text{-Im})_3]$ (5) 3
Synthesis of $[ReCl_5(\mu\text{-MeOpch})Co(Me\text{-Im})_3]$ (6) 4
Synthesis of $[ReBr_5(\mu\text{-EtOpch})Co(Me\text{-Im})_3]$ (7) 4
Synthesis of $[ReBr_5(\mu\text{-MeOpch})Co(Et\text{-Im})_3]$ (8) 4
2. Crystallography 5
Collection and Refinement 5
Table of Halogen Bond Properties 5
Crystal Data and Structure Determination Summaries 5
Additional Figures 8
Details of adjusted unit cell volume (figure 2 of main text) 10
3. Magnetic Data 11
Magnetic Data for the Ligands (9), (10), and (11) 11
Magnetic Data for the Fe^{III} containing complexes (1) - (4) 12
Magnetic Data for the Co^{III} containing complexes (5) - (8) 13

1. Experimental Details

General Remarks

Unless otherwise stated, all reagents were obtained from commercial sources and were used as received without further purification. All reactions were carried out under aerobic conditions. Elemental analyses (CHN) were performed using an Elementar Vario EL Analyzer. FTIR spectra were measured as KBr pellets over 4000-400 cm^{-1} on a Perkin Elmer *Spectrum One* spectrometer. Magnetic susceptibility data (2-300K) were collected on powdered polycrystalline samples on a Quantum Design MPMS-XL SQUID magnetometer under an applied magnetic field of 0.1 T (unless otherwise stated). All data were corrected from the sample holder contribution and the diamagnetism of the samples estimated from Pascal's constants.

Synthesis of the $(NBu_4)[Re^{IV}Br_5(H_2MeOpch)]$ ligand (9)

$(NBu_4)_2ReBr_6$ (1.61 g, 1.40 mmol) and $H_2MeOpch$ (2.29 g, 8.40 mmol) were dissolved in a mixture of 2-propanol/acetone (2:1, v/v, 150 mL) and heated to 75 °c for 2 hours. After cooling to room temperature, the solvent volume was reduced to ~ 80 mL and the orange precipitate collected via filtration and dried *in vacuo*. Yield: 1.34 g (87%). $C_{29}H_{48}Br_5N_5O_3Re$ (1098.44 g mol^{-1}): Calculated C: 31.7%, H: 4.4%, N: 6.4%, Found: C: 31.0%, H: 4.3%, N: 6.1%. IR (ν cm^{-1}): 2960 (m), 2932.34 (w), 2872.34 (w), 1683.21 (s), 1608,74 (w), 1528.71 (m), 1462.46 (s), 1406.72 (m), 1377.19 (m), 1353.85 (m), 1244.76 (vs), 1148.87 (s), 1077.4 (s), 968.01 (m), 952.43 (m), 905.74 (m), 881.17 (m), 781.71 (m), 629.96 (w), 482.05 (m). UV/Vis: (MeCN) (λ nm) = 201, 220, 305, 339, 357 (sh), 590.

Synthesis of the $(NBu_4)[Re^{IV}Cl_5(H_2MeOpch)]$ ligand (10)

$(NBu_4)_2ReCl_6$ (0.088 g, 0.10 mmol) and $H_2MeOpch$ (0.163 mg, 0.6 mmol) were dissolved in a mixture of 2-propanol/acetone (2:1, v/v, 30 mL) and heated to 75 °c for 6 hours. After cooling to room temperature, the solution was filtered and left to stand under an inert atmosphere. After six days orange needles were collected via filtration and dried *in vacuo*. Yield: 0.067 g (67%). $C_{29}H_{48}Cl_5N_5O_3Re$ (997.35 g mol^{-1}): Calculated C: 42.2%, H: 6.4%, N: 7.0%, Found: C: 40.9%, H: 6.4%, N: 7.0%. IR (ν cm^{-1}): 3490.64 (vw), 2960.63 (s), 2932.34 (m), 2873.71 (m), 1682.29 (vs), 1608.93 (m), 1576.7 (w), 1533.26 (s), 1464.20 (vs), 1407.37 (m), 1377.3 (m), 1354.51 (s), 1282.71 (s), 1246.52 (vs), 1147.85 (vs), 1076.59 (s), 1024.27 (m), 952.16 (s), 906.25 (m), 881.89 (m), 835.5 (w), 817.35 (w), 783.08 (m), 739.27 (vs), 486.03 (m), 344.91 (s). UV/Vis: (MeCN) (λ nm) = 200, 221, 292, 353 (sh).

Synthesis of the $(NBu_4)[Re^{IV}Br_5(H_2EtOpch)]$ ligand (11)

$(NBu_4)_2ReBr_6$ (0.080 g, 0.07 mmol) and $H_2EtOpch$ (0.120 g, 0.42 mmol) were dissolved in a mixture of 2-propanol/acetone (2:1, v/v, 30 mL) and heated to 70 °c for 3 hours. After cooling to room temperature, the solvent volume was reduced to ~ 10 mL and the orange precipitate collected via filtration and dried *in vacuo*. Yield: 0.064 g (82%). $C_{30}H_{49}Br_5N_5O_3Re$ (1113.48 g mol^{-1}): Calculated C: 32.4%, H: 4.4%, N: 6.3%, Found: C: 32.6%, H: 4.5%, N: 6.3%. IR (ν cm^{-1}): 3298.49 (vw), 2953.33 (w), 2928.07 (w), 2868.39 (w), 1708.94 (s), 1607.47 (m), 1513.86 (s), 1461.39 (s), 1403.19 (s), 1377.04 (m), 1354.87 (m), 1255.09 (vs), 1153.09 (vs), 1111.79 (m), 1057.44 (s), 1023.05 (s), 948.36 (m), 922.13 (w), 882.87 (s), 778.03 (m), 732.34 (vs), 581.57 (s), 486.33 (s), 443.94 (w). UV/Vis: (MeCN) (λ nm) = 221, 265, 305, 339, 357 (sh).

Synthesis of $[ReBr_5(\mu\text{-}MeOpch)Fe(Me\text{-}Im)_3]$ (1)

A solution of $Fe(ClO_4)_2{\cdot}6H_20$ (7 mg, 0.02 mmol) in MeCN (5 mL) was slowly added to a solution of $(NBu_4)[ReBr_5(H_2MeOpch)]$ (9) (12 mg, 0.01 mmol) and 1-Me-Im (4 mg, 0.04 mmol) in MeCN (5 mL).

The reddish-brown clear solution was sealed and left to stand for three weeks until dark brown needle crystals were formed. Yield: 5 mg (48%). $C_{25}H_{28}Br_5FeN_{10}O_3Re$ (1158.2 g mol^{-1}): Calculated C: 25.9%, H: 2.4%, N: 12.1%, Found: C: 26.5%, H: 2.7%, N: 12.7%. IR (ν cm^{-1}): 3123.92 (w), 1589.64 (m), 1536.67(m), 1515.52 (m), 1466.97 (w), 1431.21 (s), 1349.79 (s), 1287.89 (m), 1252.10 (s), 1231.95 (m),1213.83 (m), 1156.22 (m), 1087.77 (vs), 1020.01 (w), 969.69 (w), 948.75 (m), 923.14 (w), 825.53 (w), 785.56 (w), 750.87 (vs), 655.89 (s), 618.26 (m), 584.20 (s), 509.16 (w), 416.01 (s), 370.45 (m). UV/Vis: (MeCN) (λ nm) = 216, 281, 337, 350, 360.

Synthesis of [ReCl$_5$(μ-MeOpch)Fe(Me-Im)$_3$] (2)

A solution of $Fe(ClO_4)_2 \cdot 6H_20$ (7 mg, 0.02 mmol) in MeCN (5 mL) was slowly added to a solution of (NBu_4)[ReCl$_5$(H_2MeOpch)] (10) (12 mg, 0.01 mmol) and 1-Me-Im (3 mg, 0.03 mmol) in MeCN (5 mL). The reddish-brown clear solution was sealed and left to stand for eight days until dark brown needle crystals were formed. Yield: 6 mg (54%). $C_{25}H_{28}Cl_5FeN_{10}O_3Re$ (1158.2 g mol^{-1}): Calculated C: 32.1%, H: 3.0%, N: 15.0%, Found: C: 31.2%, H: 3.0%, N: 14.7%. IR (ν cm^{-1}): 2959.82 (vw), 2932.16 (vw),2834.89 (vw), 1599.07 (s), 1548.96 (s), 1509.87 (m), 1436.95 (s), 1354.18 s), 1298.48 (m), 1242.86 (s), 1219.02 (vs), 1157.91 (s), 1081.29 (m), 1020.25 (w), 971.61 (w), 921.75 (w), 856.03 (m), 741.14 (vs), 629.47 (w), 552.75 (m), 492.86 (w), 442.83 (m), 404.69 (m), 360.83 (w). UV/Vis: (MeCN) (λ nm) = 205, 282, 326 (sh), 338, 381, 477.

Synthesis of [ReBr$_5$(μ-EtOpch)Fe(Me-Im)$_3$] (3)

A solution of $Fe(ClO_4)_2 \cdot 6H_20$ (35 mg, 0.12 mmol) in MeCN (10 mL) was slowly added to a solution of (NBu_4)[ReBr$_5$(H_2EtOpch)] (11) (60 mg, 0.06 mmol) and 1-Me-Im (20 mg, 0.24 mmol) in MeCN (10 mL). The reddish-brown clear solution was sealed and left to stand for four days until dark brown block crystals were formed. Yield: 5 mg (48%). $C_{26}H_{31}Br_5FeN_{10}O_3Re$ (1172.67 g mol^{-1}): Calculated C: 26.6%, H: 2.6%, N: 11.9%, Found: C: 26.1%, H: 2.4%, N: 12.1%. IR (ν cm^{-1}): 3117.69 (w), 1606.41 (w), 1588.29 (m), 1532.37 (w), 1433.95 (m), 1391.48 (m), 1350.72 (m), 1280.02 (w), 1254.65 (s), 1211.11 (m), 1157.54 (m), 1086.37 (vs), 1023.52 (m), 946.05 (m), 901.05 (w), 844.64 (m), 783.74 (w), 741.72 (vs), 655.11 (s), 616.44 (m), 569.65 (m), 510.62 (m), 463.04 (w), 432.02 (m), 365.91 (w). UV/Vis: (MeCN) (λ nm) = 195, 208, 221, 307, 335, 355, 388 (sh).

Synthesis of [ReBr$_5$(μ-MeOpch)Fe(Et-Im)$_3$] (4)

A solution of $Fe(ClO_4)_2 \cdot 6H_20$ (22 mg, 0.06 mmol) in MeCN (10 mL) was slowly added to a solution of (NBu_4)[ReBr$_5$(H_2MeOpch)] (9) (60 mg, 0.06 mmol) and 1-Me-Im (12 mg, 0.12 mmol) in MeCN (15 mL). The reddish-brown clear solution was sealed and left to stand for one day until dark brown block crystals were formed. Yield: 31 mg (43%). $C_{28}H_{34}Br_5CoN_{10}O_3Re$ (1153.26 g mol^{-1}): Calculated C: 26.9%, H: 2.6%, N: 11.3%, Found: C: 26.6%, H: 2.7%, N: 11.3%. IR (ν cm^{-1}): 3118.97 (w), 2976.85 (vw), 934.43 (vw), 1589.94 (m), 1550.07 (m), 1531.44 (m), 1462.85 (m), 1426.59 (m), 1350.16 (s), 1278.91 (m), 1245.55 (m), 1230.54 (m), 1215.49 (m), 1182.96 (w), 1158.06 (m), 1087.85 (vs), 1020.19 (m), 958.38 (m), 945.17 (m), 838.72 (m), 798.84 (w), 744.99 (vs), 660.59 (s), 590.25 (m), 567.34 (m), 512.18 (m), 431.91 (m), 359.09 (s). UV/Vis: (MeCN) (λ nm) = 191, 209, 274 (sh), 338, 363, 392.

Synthesis of [ReBr$_5$(μ-MeOpch)Co(Me-Im)$_3$] (5)

A solution of $Fe(ClO_4)_2 \cdot 6H_20$ (7 mg, 0.02 mmol) in MeCN (5 mL) was slowly added to a solution of (NBu_4)[ReBr$_5$(H_2MeOpch)] (9) (12 mg, 0.01 mmol) and 1-Me-Im (4 mg, 0.04 mmol) in MeCN (5 mL). The reddish-brown clear solution was sealed and left to stand for three weeks until dark brown needle crystals were formed. Yield: 5 mg (48%). $C_{25}H_{28}Br_5FeN_{10}O_3Re$ (1158.2 g mol^{-1}): Calculated C: 25.9%, H: 2.4%, N: 12.1%, Found: C: 26.5%, H: 2.7%, N: 12.7%. IR (ν cm^{-1}): 3123.92 (w), 1589.64 (m), 1536.67(m), 1515.52 (m), 1466.97 (w), 1431.21 (s), 1349.79 (s), 1287.89 (m), 1252.10 (s), 1231.95 (m),1213.83 (m), 1156.22 (m), 1087.77 (vs), 1020.01 (w), 969.69 (w), 948.75 (m), 923.14 (w), 825.53 (w), 785.56 (w),

750.87 (vs), 655.89 (s), 618.26 (m), 584.20 (s), 509.16 (w), 416.01 (s), 370.45 (m). UV/Vis: (MeCN) (λ nm) = 216, 281, 337, 350, 360.

Synthesis of [$ReCl_5$(μ-MeOpch)Co(Me-Im)$_3$] (6)

A solution of $Co(ClO_4)_2$·$6H_20$ (5 mg, 0.01 mmol) in EtOH (5 mL) was slowly added to a solution of (NBu_4)[$ReCl_5$(H_2MeOpch)] (10) (12 mg, 0.01 mmol) and 1-Me-Im (3 mg, 0.03 mmol) in MeCN (5 mL). The reddish-brown clear solution was sealed and left to stand for one week until dark brown block crystals were formed. Yield: 6 mg (53%). $C_{25}H_{28}Cl_5CoN_{10}O_3Re$ (998.97 g mol^{-1}): Calculated C: 32.0%, H: 3.0%, N: 14.9%, Found: C: 31.9%, H: 2.9%, N: 15.0%. IR (ν cm^{-1}): 3124.23 (m), 1619.89 (w), 1596.86 (w), 1540.02 (s), 1467.91 (m), 1438.98 (s), 1342.99 (m), 1281.38 (w), 1243.16 (s), 1220.03 (s), 171.47 (m), 1154.42 (m), 1100.26 (vs), 1073.79 (s), 1023.86 (w), 982.06 (w), 960.28 (w), 937.12 (w), 857.99 (m), 839.21 (m), 739.99 (vs), 658.66 (m), 619.79 (s), 483.14 (m), 443.78 (vw), 346.04 (m). UV/Vis: (MeCN) (λ nm) = 198, 205, 250, 277, 338, 439.

Synthesis of [$ReBr_5$(μ-EtOpch)Co(Me-Im)$_3$] (7)

A solution of $Co(ClO_4)_2$·$6H_20$ (7 mg, 0.02 mmol) in EtOH (10 mL) was slowly added to a solution of (NBu_4)[$ReBr_5$(H_2EtOpch)] (11) (13 mg, 0.01 mmol) and 1-Me-Im (3 mg, 0.04 mmol) in MeCN (10 mL). The reddish-brown clear solution was sealed and left to stand for one day until dark brown block crystals were formed. Yield: 8 mg (55%). $C_{26}H_{31}Br_{Co}oN_{10}O_3Re$ (1176.26 g mol^{-1}): Calculated C: 26.6%, H: 2.7%, N: 12.2%, Found: C: 26.7%, H: 2.7%, N: 12.2%. IR (ν cm^{-1}): 3113.99 (w), 1613.82 (m), 1592.62 (m), 1537.68 (s), 1462.75 (m), 1436.98 (m), 1417.29 (m), 1391.40 (w), 1341.99 (w), 1281.69 (w), 1240.46 (s), 1212.97 (s), 1175.62 (w), 1152.86 (m), 1091.30 (vs), 1024.52 (m), 937.60 (w), 895.09 (w), 834.93 (m), 779.17 (w), 758.68 (vs), 738.02 (vs), 661.52 (s), 617.78 (s), 540.25 (w), 480.37 (m), 451.54 (w), 351.88 (s). UV/Vis: (MeCN) (λ nm) = 186, 212, 247, 307, 334, 355, 389 (sh), 422.

Synthesis of [$ReBr_5$(μ-MeOpch)Co(Et-Im)$_3$] (8)

A solution of $Co(ClO_4)_2$·$6H_20$ (5 mg, 0.01 mmol) in MeCN (10 mL) was slowly added to a solution of (NBu_4)[$ReBr_5$(H_2MeOpch)] (9) (12 mg, 0.01 mmol) and 1-Me-Im (3 mg, 0042 mmol) in MeCN (10 mL). The reddish-brown clear solution was sealed and left to stand for three weeks until dark brown block crystals were formed. Yield: 7 mg (62%). $C_{28}H_{33}Br_5FeN_{10}O_3Re$ (1202.26 g mol^{-1}): Calculated C: 28.0%, H: 2.8%, N: 11.7%, Found: C: 28.0%, H: 3.0%, N: 11.9%. IR (ν cm^{-1}): 3130.42 (w), 1619.41 (w), 1595.53 (m), 1529.95 (s), 1466.28 (m), 1437.65 (m), 1402.98 (w), 1378.53 (w), 1344.97 (m), 1289.18 (w), 1241.47 (s), 1216.45 (s), 1168.22 (m), 1092.77 (vs), 1031.92 (m), 960.98 (w), 937.2 (m), 837.08 (m), 739.79 (vs), 663.25 (s), 623.94 (m), 529.48 (vw), 483.76 (m), 357.56 (w). UV/Vis: (MeCN) (λ nm) = 197, 210, 250, 281, 341, 358, 433.

2. Crystallography

Collection and Refinement

Data was either measured at the SCD beamline of the ANKA Synchrotron light source, Karlsruhe, on a Bruker SMART Apex diffractometer using Si-monochromated radiation with λ = 0.80000 Å, or on a Rigaku Oxford Diffraction SuperNova E diffractometer equipped with Cu-Ka and Mo-Ka microfocus sources. All data were corrected semi-empirically for absorption. Structures were solved using SHELXT [G.M. Sheldrick, Acta Cryst. A71, 3-8 (2015)] and full-matrix least-squares refinement with anisotropic thermal parameters was carried out using SHELXL [G.M. Sheldrick, Acta Cryst. C71, 3-8 (2015)]. For further details of the refinements of the structures, see _refine_special_details in the individual CIFs. Crystallographic data for the structures in this paper have been deposited with the Cambridge Crystallographic Data Centre as supplementary publication nos. CCDC 1978010-1978029. Copies of the data can be obtained, free of charge, from https://www.ccdc.cam.ac.uk/structures/

Table of Halogen Bond Properties

Crystal-ID	X	M^{III}	Lattice solvent	R-im	R-lig	T / K	Re-X···X (Å)	Re-X-X (◦)	N_c (R_{XB})
1w_100	Br	Fe	2MeCN	Me	OMe	100	4,663	109.92/130.94	1,260
1w_230	Br	Fe	2MeCN	Me	OMe	230	4,587	110.26/131.37	1,240
1d_100	Br	Fe	no	Me	OMe	100	3,733	176.03	1,009
1d_140	Br	Fe	no	Me	OMe	140	3,742	176.13	1,011
1d_180	Br	Fe	no	Me	OMe	180	3,901	175.1	1,054
1d_230	Br	Fe	no	Me	OMe	230	3,982	174.68	1,076
1d_280	Br	Fe	no	Me	OMe	280	4,109	174.2	1,111
2w_100	Cl	Fe	2MeCN	Me	OMe	100	4,539	110.53/133.95	1,297
3w_180	Br	Fe	MeCN	Me	OEt	180	4,099	140.18/159.24	1,108
3d_180	Br	Fe	no	Me	OEt	180	**3.888**/4.599/4.644	**121.52**/168.75/167.59	**1.051**/1.243/1.255
4d_180	Br	Fe	no	Et	OMe	180	4,455	166.61	1,204
5w_180	Br	Co	MeCN·MeOH	Me	OMe	180	4.072/4.283/4.866	164.22/145.50/153.74	1.100/1.158/1.315
5d_100	Br	Co	no	Me	OMe	100	3,626	176.95	0.980
5d_230	Br	Co	no	Me	OMe	230	3,661	177.34	0.989459459
6w_180	Cl	Co	2MeCN	Me	OMe	180	4,703	110.27/131.87	1,344
7w_180	Br	Co	MeCN	Me	OEt	180	4,261	166.87	1,152
8w_180	Br	Co	MeCN·0.5MeOH	Et	OMe	180	4.411/4.945	109.94/95.94	1.192/1.336

Crystal Data and Structure Determination Summaries

1d_100: $C_{25}H_{28}Br_5FeN_{10}O_3Re$ (M =1158.17 g/mol): monoclinic, space group C2/c (no. 15), a = 24.1323(5) Å, b = 13.8406(3) Å, c = 22.3530(6) Å, β = 103.769(3)°, V = 7251.5(3) Å^3, Z = 8, T = 100(2) K, μ(CuKα) = 16.450 mm^{-1}, D_{calc} = 2.122 g/cm^3, 20097 reflections measured (7.418° ≤ 2Θ ≤ 141.674°), 6867 unique (R_{int} = 0.0452, R_{sigma} = 0.0536) which were used in all calculations. The final R_1 was 0.0445 (I > 2σ(I)) and wR_2 was 0.1208 (all data). CCDC 1978029.

1d_140: $C_{25}H_{28}Br_5FeN_{10}O_3Re$ (M =1158.17 g/mol): monoclinic, space group C2/c (no. 15), a = 24.1452(7) Å, b = 13.8798(4) Å, c = 22.3861(7) Å, β = 103.841(3)°, V = 7284.4(4) Å^3, Z = 8, T = 140(2) K, μ(CuKα) = 16.376 mm^{-1}, D_{calc} = 2.112 g/cm^3, 18082 reflections measured (7.402° ≤ 2Θ ≤ 141.71°), 6883 unique (R_{int} = 0.0405, R_{sigma} = 0.0597) which were used in all calculations. The final R_1 was 0.0441 (I > 2σ(I)) and wR_2 was 0.0884 (all data). CCDC 1978010.

1d_180: $C_{25}H_{28}Br_5FeN_{10}O_3Re$ (M =1158.17 g/mol): monoclinic, space group C2/c (no. 15), a = 24.4964(5) Å, b = 13.9397(3) Å, c = 22.4472(5) Å, β = 103.415(2)°, V = 7456.0(3) Å^3, Z = 8, T = 180(2) K, μ(CuKα) = 15.999 mm^{-1}, D_{calc} = 2.064 g/cm^3, 20971 reflections measured (7.348° ≤ 2Θ ≤ 141.68°), 7060 unique (R_{int} = 0.0416, R_{sigma} = 0.0452) which were used in all calculations. The final R_1 was 0.0431 (I > 2σ(I)) and wR_2 was 0.1155 (all data). CCDC 1978011.

1d_230: $C_{25}H_{28}Br_5FeN_{10}O_3Re$ (*M* =1158.17 g/mol): monoclinic, space group C2/c (no. 15), *a* = 24.6698(5) Å, *b* = 13.9938(3) Å, *c* = 22.5024(6) Å, *β* = 103.306(2)°, *V* = 7559.8(3) Å^3, *Z* = 8, *T* = 230(2) K, μ(CuKα) = 15.779 mm^{-1}, D_{calc} = 2.035 g/cm^3, 21356 reflections measured (7.312° ≤ 2Θ ≤ 141.834°), 7176 unique (R_{int} = 0.0418, R_{sigma} = 0.0430) which were used in all calculations. The final R_1 was 0.0392 (I > 2σ(I)) and wR_2 was 0.1033 (all data). CCDC 1978021.

1d_280: $C_{25}H_{28}Br_5FeN_{10}O_3Re$ (*M* =1158.17 g/mol): monoclinic, space group C2/c (no. 15), *a* = 24.7548(9) Å, *b* = 13.9993(5) Å, *c* = 22.5547(9) Å, *β* = 103.357(4)°, *V* = 7604.9(5) Å^3, *Z* = 8, *T* = 280(2) K, μ(CuKα) = 15.685 mm^{-1}, D_{calc} = 2.023 g/cm^3, 21146 reflections measured (7.304° ≤ 2Θ ≤ 141.882°), 7221 unique (R_{int} = 0.0451, R_{sigma} = 0.0591) which were used in all calculations. The final R_1 was 0.0483 (I > 2σ(I)) and wR_2 was 0.0885 (all data). CCDC 1978019.

1w_100: $C_{29}H_{35}Br_5FeN_{12}O_{3.5}Re$ (*M* =1249.29 g/mol): monoclinic, space group C2/c (no. 15), *a* = 24.2298(14) Å, *b* = 14.2081(8) Å, *c* = 23.6869(10) Å, *β* = 102.321(5)°, *V* = 7966.6(7) Å^3, *Z* = 8, *T* = 100.00(10) K, μ(CuKα) = 15.060 mm^{-1}, D_{calc} = 2.083 g/cm^3, 16388 reflections measured (7.256° ≤ 2Θ ≤ 141.49°), 7529 unique (R_{int} = 0.0517, R_{sigma} = 0.0783) which were used in all calculations. The final R_1 was 0.0650 (I > 2σ(I)) and wR_2 was 0.1530 (all data). CCDC 1978014.

1w_230: $C_{29}H_{35}Br_5FeN_{12}O_{3.5}Re$ (*M* =1249.29 g/mol): monoclinic, space group C2/c (no. 15), *a* = 24.5066(13) Å, *b* = 14.4369(7) Å, *c* = 23.7448(9) Å, *β* = 102.709(4)°, *V* = 8195.1(7) Å^3, *Z* = 8, *T* = 230.00(10) K, μ(CuKα) = 14.640 mm^{-1}, D_{calc} = 2.025 g/cm^3, 16285 reflections measured (7.152° ≤ 2Θ ≤ 141.906°), 7709 unique (R_{int} = 0.0370, R_{sigma} = 0.0543) which were used in all calculations. The final R_1 was 0.0517 (I > 2σ(I)) and wR_2 was 0.1293 (all data). CCDC 1978028.

2w_100: $C_{29}H_{34}Cl_5FeN_{12}O_3Re$ (*M* =1017.98 g/mol): monoclinic, space group C2/c (no. 15), *a* = 24.712(2) Å, *b* = 14.0875(14) Å, *c* = 23.063(2) Å, *β* = 102.293(1)°, *V* = 7845.0(13) Å^3, *Z* = 8, *T* = 100(2) K, μ(0.85366 Å) = 6.153 mm^{-1}, D_{calc} = 1.724 g/cm^3, 28695 reflections measured (4.342° ≤ 2Θ ≤ 65.664°), 7784 unique (R_{int} = 0.0285, R_{sigma} = 0.0261) which were used in all calculations. The final R_1 was 0.0398 (I > 2σ(I)) and wR_2 was 0.1104 (all data). CCDC 1978017.

3d_180: $C_{26}H_{30}Br_5FeN_{10}O_3Re$ (*M* =1172.20 g/mol): monoclinic, space group $P2_1/n$ (no. 14), *a* = 14.3833(11) Å, *b* = 15.8410(11) Å, *c* = 17.2008(15) Å, *β* = 113.637(6)°, *V* = 3590.3(5) Å^3, *Z* = 4, *T* = 180(2) K, μ(GaKα) = 11.395 mm^{-1}, D_{calc} = 2.169 g/cm^3, 21251 reflections measured (6.884° ≤ 2Θ ≤ 107.812°), 6450 unique (R_{int} = 0.1851, R_{sigma} = 0.2805) which were used in all calculations. The final R_1 was 0.0541 (I > 2σ(I)) and wR_2 was 0.1205 (all data). CCDC 1978023.

3w_180: $C_{28}H_{33}Br_5FeN_{11}O_3Re$ (*M* =1213.25 g/mol): monoclinic, space group $P2_1/n$ (no. 14), *a* = 23.1592(3) Å, *b* = 13.8258(2) Å, *c* = 24.8952(4) Å, *β* = 103.135(1)°, *V* = 7762.8(2) Å^3, *Z* = 8, *T* = 180(2) K, μ(CuKα) = 15.412 mm^{-1}, D_{calc} = 2.076 g/cm^3, 29732 reflections measured (7.292° ≤ 2Θ ≤ 142.04°), 14712 unique (R_{int} = 0.0307, R_{sigma} = 0.0443) which were used in all calculations. The final R_1 was 0.0343 (I > 2σ(I)) and wR_2 was 0.0804 (all data). CCDC 1978018.

4d_180: $C_{28}H_{34}Br_5FeN_{10}O_3Re$ (*M* =1200.25 g/mol): monoclinic, space group C2/c (no. 15), *a* = 25.2991(12) Å, *b* = 14.2403(10) Å, *c* = 22.9345(10) Å, *β* = 102.757(5)°, *V* = 8058.6(8) Å^3, *Z* = 8, *T* = 180(2) K, μ(CuKα) = 14.830 mm^{-1}, D_{calc} = 1.979 g/cm^3, 25588 reflections measured (7.166° ≤ 2Θ ≤ 141.424°), 7633 unique (R_{int} = 0.0509, R_{sigma} = 0.0642) which were used in all calculations. The final R_1 was 0.0844 (I > 2σ(I)) and wR_2 was 0.2455 (all data). CCDC 1978012.

5d_100: $C_{25.5}H_{28.75}Br_5CoN_{10.25}O_3Re$ (*M* =1171.52 g/mol): monoclinic, space group C2/c (no. 15), *a* = 23.9170(6) Å, *b* = 13.7971(3) Å, *c* = 22.3230(5) Å, *β* = 103.813(2)°, *V* = 7153.2(3) Å^3, *Z* = 8, *T* = 100(2) K, μ(CuKα) = 17.066 mm^{-1}, D_{calc} = 2.176 g/cm^3, 32697 reflections measured (7.452° ≤ 2Θ ≤ 143.454°), 6886 unique (R_{int} = 0.0315, R_{sigma} = 0.0313) which were used in all calculations. The final R_1 was 0.0476 (I > 2σ(I)) and wR_2 was 0.1127 (all data). CCDC 1978013.

5d_230: $C_{25.5}H_{28.75}Br_5CoN_{10.25}O_3Re$ (*M* =1171.52 g/mol): monoclinic, space group C2/c (no. 15), *a* = 24.0411(7) Å, *b* = 13.8865(3) Å, *c* = 22.4642(6) Å, *β* = 103.895(3)°, *V* = 7280.1(3) Å^3, *Z* = 8, *T* = 230(2) K, μ(CuKα) = 16.769 mm^{-1}, D_{calc} = 2.138 g/cm^3, 24167 reflections measured (7.408° ≤ 2Θ ≤ 143.394°), 6976 unique (R_{int} = 0.0313, R_{sigma} = 0.0411) which were used in all calculations. The final R_1 was 0.0456 (I > 2σ(I)) and wR_2 was 0.0963 (all data). CCDC 1978022.

5w_180: $C_{28}H_{35}Br_5CoN_{11}O_4Re$ (*M* =1234.35 g/mol): monoclinic, space group C2/c (no. 15), *a* = 23.8563(3) Å, *b* = 13.8521(2) Å, *c* = 23.8458(3) Å, *β* = 100.286(2)°, *V* = 7753.43(18) Å^3, *Z* = 8, *T* = 180(2) K, μ(CuKα) = 15.813 mm^{-1}, D_{calc} = 2.115 g/cm^3, 26265 reflections measured (7.41° ≤ 2Θ ≤ 142.022°), 7406 unique (R_{int} = 0.0324, R_{sigma} = 0.0282) which were used in all calculations. The final R_1 was 0.0349 (I > 2σ(I)) and wR_2 was 0.0836 (all data). CCDC 1978015.

6w_180: $C_{29}H_{34}Cl_5CoN_{12}O_3Re$ (*M* =1021.06 g/mol): monoclinic, space group C2/c (no. 15), *a* = 24.7366(12) Å, *b* = 14.3511(9) Å, *c* = 22.5947(7) Å, *β* = 102.360(4)°, *V* = 7835.1(7) Å^3, *Z* = 8, *T* = 180(2) K, μ(CuKα) = 12.806 mm^{-1}, D_{calc} = 1.731 g/cm^3, 15724 reflections measured (7.164° ≤ 2Θ ≤ 142.352°), 7458 unique (R_{int} = 0.0609, R_{sigma} = 0.0904) which were used in all calculations. The final R_1 was 0.0663 (I > 2σ(I)) and wR_2 was 0.1686 (all data). CCDC 1978016.

7w_180: $C_{28}H_{33}Br_5CoN_{11}O_3Re$ (*M* =1216.33 g/mol): monoclinic, space group P2$_1$/n (no. 14), *a* = 23.2303(5) Å, *b* = 13.7099(3) Å, *c* = 24.4859(5) Å, *β* = 102.010(2)°, *V* = 7627.7(3) Å^3, *Z* = 8, *T* = 180(2) K, μ(CuKα) = 16.042 mm^{-1}, D_{calc} = 2.118 g/cm^3, 29554 reflections measured (7.382° ≤ 2Θ ≤ 142.124°), 14482 unique (R_{int} = 0.0524, R_{sigma} = 0.0744) which were used in all calculations. The final R_1 was 0.0603 (I > 2σ(I)) and wR_2 was 0.1506 (all data). CCDC 1978027.

8w_180: $C_{30.5}H_{39}Br_5CoN_{11}O_{3.5}Re$ (*M* =1260.40 g/mol): monoclinic, space group C2/c (no. 15), *a* = 24.2829(5) Å, *b* = 13.9421(2) Å, *c* = 23.8860(4) Å, *β* = 99.584(4)°, *V* = 7973.8(3) Å^3, *Z* = 8, *T* = 180(2) K, μ(CuKα) = 15.384 mm^{-1}, D_{calc} = 2.100 g/cm^3, 27850 reflections measured (7.338° ≤ 2Θ ≤ 141.986°), 7629 unique (R_{int} = 0.0307, R_{sigma} = 0.0266) which were used in all calculations. The final R_1 was 0.0449 (I > 2σ(I)) and wR_2 was 0.1099 (all data). CCDC 1978024.

9: $C_{35}H_{64}Br_5N_5O_5Re$ (*M* =1220.66 g/mol): monoclinic, space group C2/c (no. 15), *a* = 32.1559(4) Å, *b* = 11.2083(1) Å, *c* = 27.4170(4) Å, *β* = 109.270(2)°, *V* = 9327.8(2) Å^3, *Z* = 8, *T* = 149.99(10) K, μ(CuKα) = 10.444 mm^{-1}, D_{calc} = 1.738 g/cm^3, 31497 reflections measured (6.83° ≤ 2Θ ≤ 143.682°), 9052 unique (R_{int} = 0.0273, R_{sigma} = 0.0251) which were used in all calculations. The final R_1 was 0.0276 (I > 2σ(I)) and wR_2 was 0.0717 (all data). CCDC 1978025.

10: $C_{35}H_{64}Cl_5N_5O_5Re$ (*M* =998.36 g/mol): monoclinic, space group C2/c (no. 15), *a* = 32.1928(6) Å, *b* = 10.9285(2) Å, *c* = 27.3411(5) Å, *β* = 109.697(2)°, *V* = 9056.3(3) Å^3, *Z* = 8, *T* = 180(2) K, μ(CuKα) = 8.301 mm^{-1}, D_{calc} = 1.464 g/cm^3, 39371 reflections measured (8.602° ≤ 2Θ ≤ 142.076°), 8685 unique (R_{int} = 0.0430, R_{sigma} = 0.0290) which were used in all calculations. The final R_1 was 0.0461 (I > 2σ(I)) and wR_2 was 0.1283 (all data). CCDC 1978020.

11: $C_{33}H_{58}Br_5N_5O_4Re$ (M =1174.59 g/mol): triclinic, space group P$\bar{1}$ (no. 2), a = 11.4661(5) Å, b = 14.3132(6) Å, c = 15.2779(4) Å, α = 71.154(3)°, β = 71.899(3)°, γ = 74.960(4)°, V = 2219.93(16) Å^3, Z = 2, T = 100(2) K, μ(MoKα) = 7.274 mm^{-1}, D_{calc} = 1.757 g/cm^3, 18039 reflections measured (4.432° ≤ 2Θ ≤ 58.232°), 10099 unique (R_{int} = 0.0259, R_{sigma} = 0.0460) which were used in all calculations. The final R_1 was 0.0349 (I > 2σ(I)) and wR_2 was 0.0789 (all data). CCDC 1978026.

For further details of the refinements of the structures, see _refine_special_details in the individual CIFs. Crystallographic data for the structures in this paper have been deposited with the Cambridge Crystallographic Data Centre as supplementary publication nos. CCDC 1978010-1978029. Copies of the data can be obtained, free of charge, from https://www.ccdc.cam.ac.uk/structures/

Additional Figures

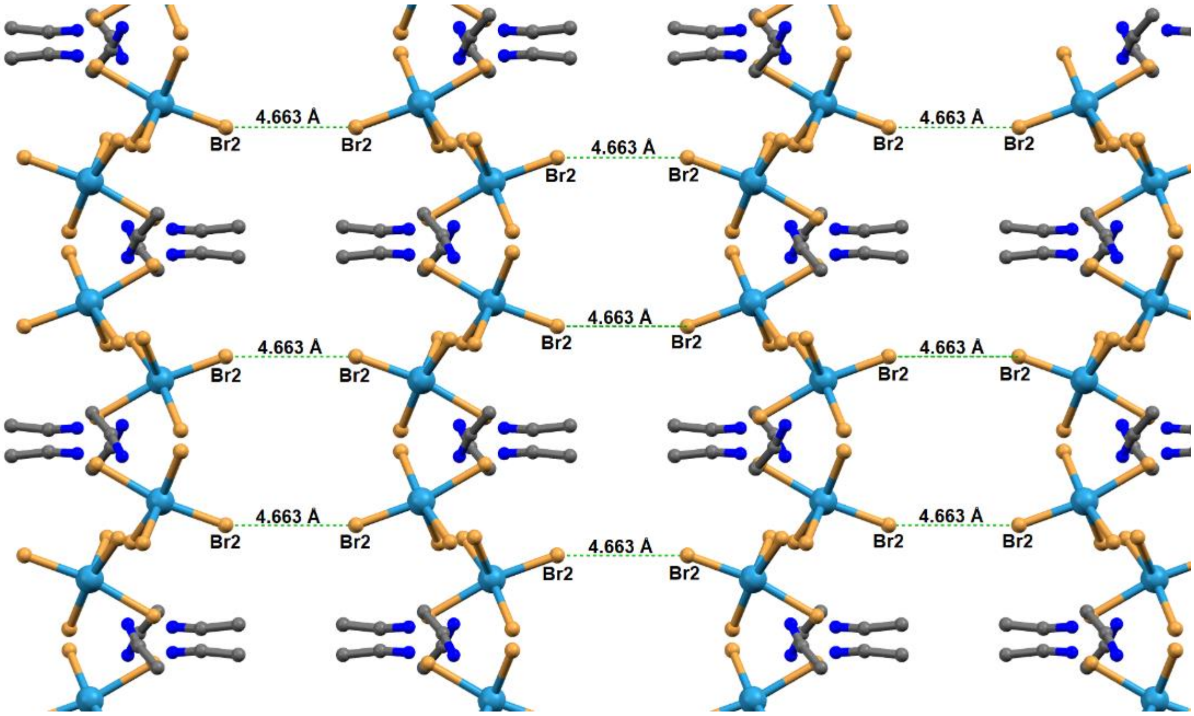

*Figure 1 - Packing arrangement of the solvated compound (**1**)w_100 demonstrating the weak long-range halogen···halogen interaction (shown down c-axis). Measured at 100 K*

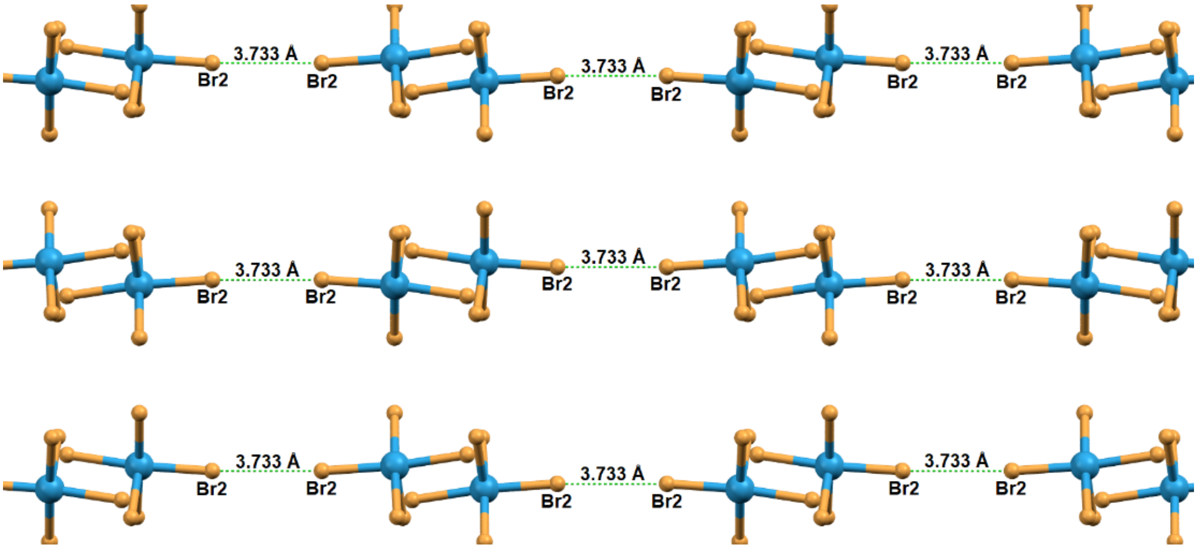

Figure 2 - Packing arrangement of the desolvated compound (1)d_100 showing the rearrangement to produce significantly more favourable halogen···halogen interactions (shown down c-axis). Measured at 100 K.

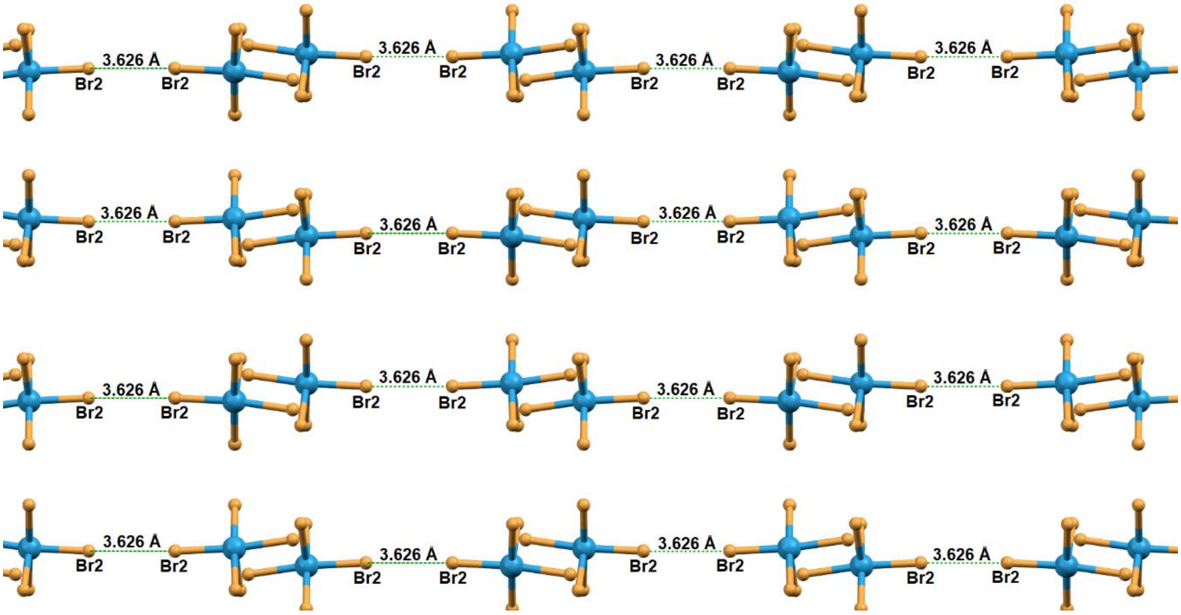

Figure 3 - Packing arrangement of the desolvated Co^{III} compound (5)d_100 showing the same packing interaction (shown down c-axis) as observed for the Fe^{III} complex. Measured at 100 K.

Details of adjusted unit cell volume (figure 2 of main text)

When looking at the unit cell volume of crystalline SCO materials across a range of temperatures transitions can clearly be followed, however, by normalising the volume to account for thermal expansion transitions becomes more defined. With complex (**1**) we investigated the analogous and isostructural Co^{III} compound (**5**), which remains LS across all measured ranges, to gain a baseline for thermal expansion. At 100 K **(5d_100)** the unit cell volume is 7153 Å^3, upon heating by 130 K to a temperature of 230 K **(5d_230)** we see expansion to 7280 Å^3, which is a difference of 127 Å^3 or ~1 Å^3 K^{-1}. By subtracting this from the changes in unit cell volume observed for (**1**) we see a much cleaner fit, particularly for the values after 220K where the volume continues to change due to thermal expansion, but the SCO event has finished (see below).

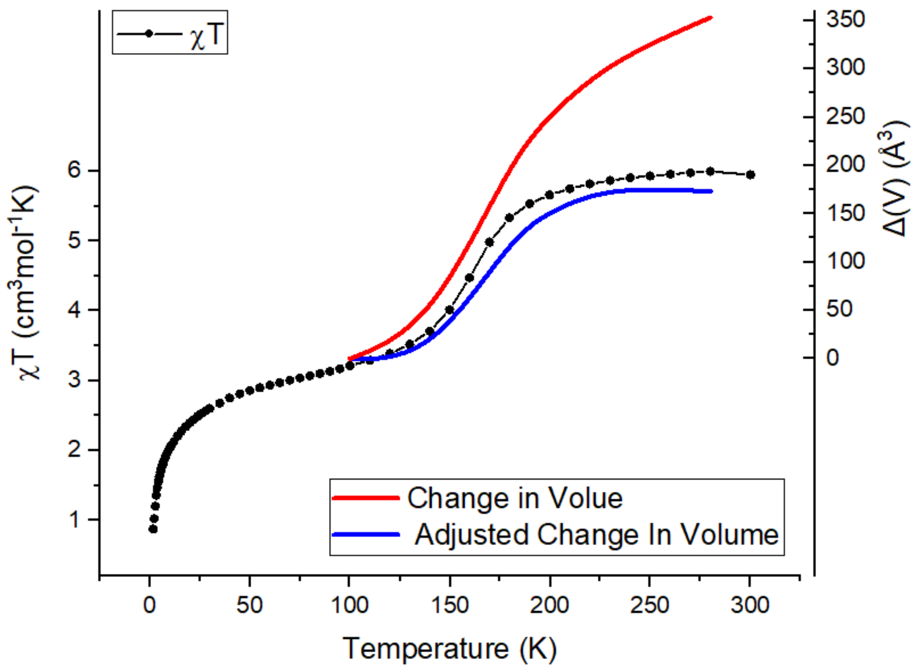

*Figure 4 – χT plot with overlaid unit cell volume and adjusted unit cell volume for complex (**1**) showing the significantly improved fit when a correction of 1 Å^3 K^{-1} is applied to the raw change in unit cell volume.*

3. Magnetic Data

Magnetic Data for the Ligands (9), (10), and (11)

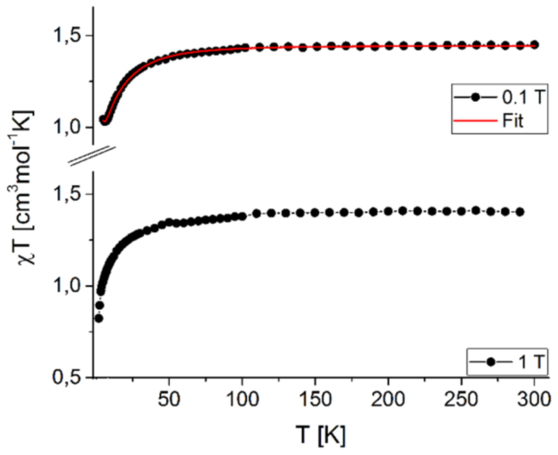

Figure 5 - χT vs T for the mononuclear ligand (9). Data collected under an applied DC field of 0.1 T.

The χT product of the mononuclear compound (9) at 300 K and an applied field of 0.1 T is 1.45 cm^3 K mol^{-1}. This is the expected value for a magnetically isolated Re^{IV} complex with a spin ground state of 3/2 and a g-factor between 1.7 and 1.9. The χT product remains near constant down to 50 K where the value drops to 0.99 cm^3 K mol^{-1} at 4 K. Interestingly the increase to 1.05 cm^3 K mol^{-1} as the temperature was further decreased to 1.8 may indicate weak ferromagnetism produced by spin canting phenomena. Collecting the data again with an applied field of 1 T (up from 0.1 t) supresses a possible canted structure.

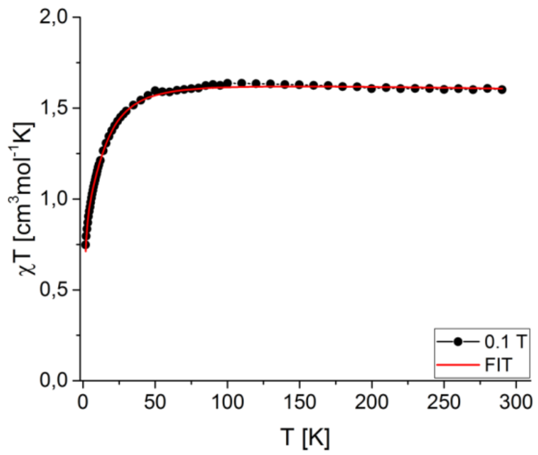

Figure 6 - χT vs T for the mononuclear ligand (10). Data collected under an applied DC field of 0.1 T.

The χT product of the mononuclear compound (10) at 300 K and an applied field of 0.1 T is 1.60 cm^3 K mol^{-1}. This is the expected value for a magnetically isolated Re^{IV} complex with a spin ground state of 3/2 and a g-factor between 1.7 and 1.9. The χT product remains near constant down to 50 K where the value drops to 0.75 cm^3 K mol^{-1} at 1.8 K. The drastic decrease in the xT value is attributed to zero field splitting of the Re^{IV} complex and potential weak antiferromagnetic interactions between metal centers.

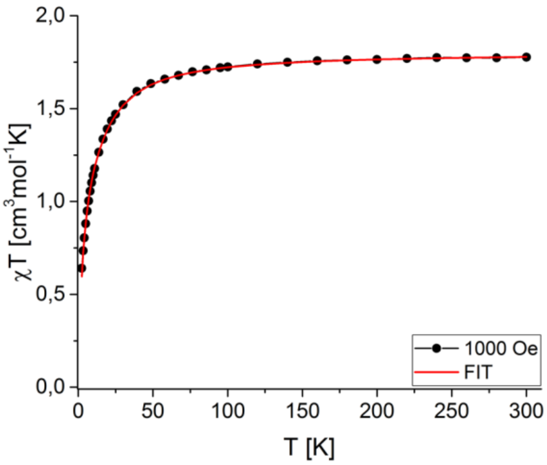

Figure 7 - χT vs T for the mononuclear ligand (11). Data collected under an applied DC field of 0.1 T.

The χT product of the mononuclear compound (9) at 300 K and an applied field of 0.1 T is 1.77 cm^3 K mol^{-1}. This is the expected value for a magnetically isolated Re^{IV} complex with a spin ground state of 3/2 and a g-factor between 1.5 and 1.9. The χT product remains near constant down to 50 K where the value drops to 0.64 cm^3 K mol^{-1} at 1.8 K. The plot χ against T shows no maximum which is infers only weak antiferromagnetic interactions are occurring. The drastic decrease in the xT value is attributed to zero field splitting of the Re^{IV} complex and potential weak antiferromagnetic interactions between metal centers.

Magnetic Data for the Fe^{III} containing complexes (1) - (4)

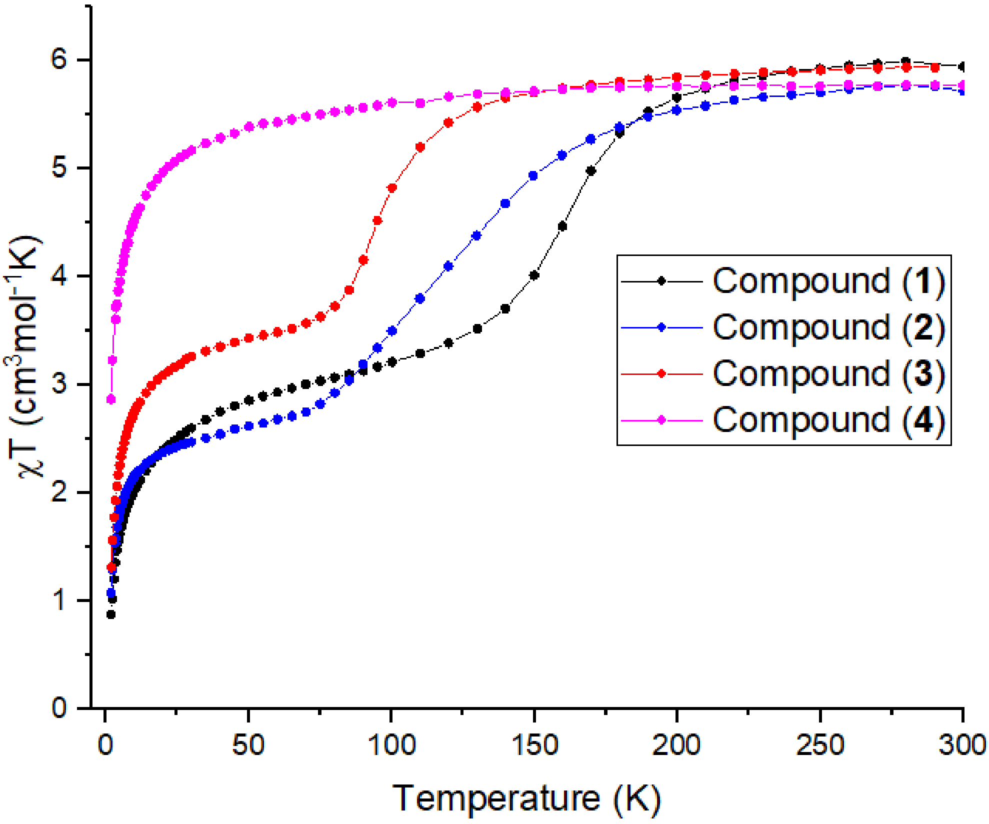

Figure 8 – Larger version of figure 4 from the paper, showing χT vs T for complexes (1) - (4). Data collected under an applied DC field of 0.1 T.

For further details of the magnetic properties of these compounds please see the main text.
The % low spin state is approximated using the following equation:

$$\frac{(X_S - X_{Re}) - (X_F - X_{Re})}{(X_S - X_{Re}) - 0.375}$$

Where X_S is the maximum χT value, X_{Re} is the χT value for the Re complexes, and X_F is the χT value at the end of the spin transition. The 0.375 $cm^3\ K\ mol^{-1}$ value is that expected for a magnetically isolated Fe^{III} in the low spin state.

This equation corrects for the contribution from the Re^{IV} spin carrier and assumes no interaction between the Fe^{III} and Re^{IV} centers.

Magnetic Data for the Co^{III} containing complexes (5) - (8)

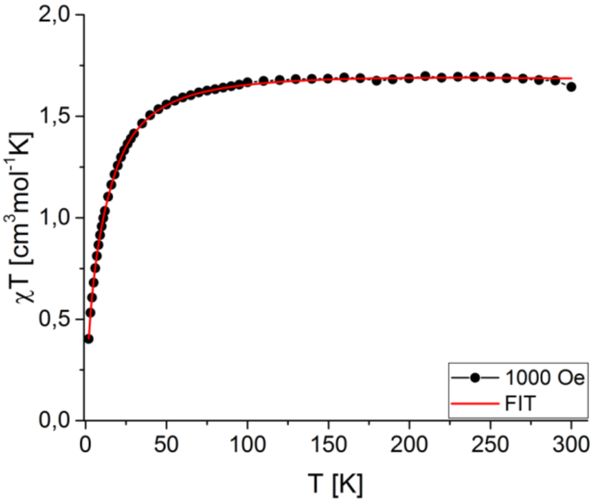

Figure 9 - χT vs T for the Co^{III}-Re^{IV} complex (5). Data collected under an applied DC field of 0.1 T.

The χT product of the Co^{III}-Re^{IV} compound (5) at 300 K and an applied field of 0.1 T is 1.64 cm^3 K mol^{-1}. This is the expected value for a magnetically isolated Re^{IV} complex with a spin ground state of 3/2 and a g-factor between 1.8 and 1.9, with a low spin (S = 0) Co^{III} ion. The χT product remains near constant down to 50 K where the value drops to 0.40 cm^3 K mol^{-1} at 1.8 K. The drop below 1.00 cm^3 K mol^{-1} is due to the zero-field splitting of the Re^{IV} ion and the existence of these interactions.

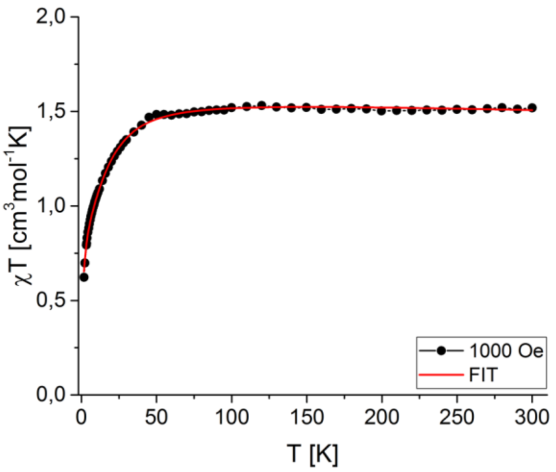

Figure 10 - χT vs T for the C^{oIII}-Re^{IV} complex (6). Data collected under an applied DC field of 0.1 T.

The χT product of the Co^{III}-Re^{IV} compound (6) at 300 K and an applied field of 0.1 T is 1.53 cm^3 K mol^{-1}. This is the expected value for a magnetically isolated Re^{IV} complex with a spin ground state of 3/2 and a g-factor between 1.8 and 1.9, with a low spin (S = 0) Co^{III} ion. The χT product remains near constant down to 50 K where the value drops to 0.38 cm^3 K mol^{-1} at 1.8 K. The drastic decrease in the xT value is attributed to zero field splitting of the Re^{IV} complex and potential weak antiferromagnetic interactions between metal centers. The drop below 1.00 cm3 K mol-1 indicates the existence of these interactions.

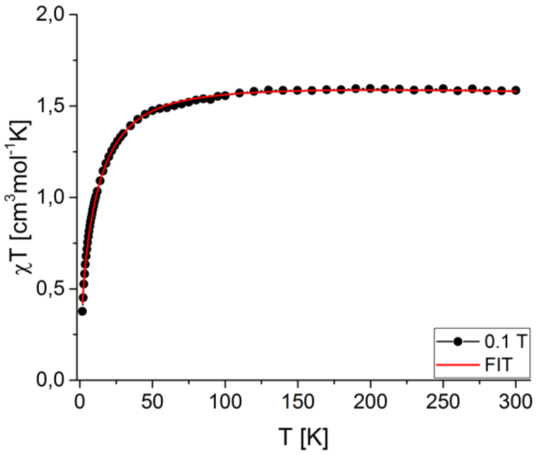

Figure 11 - χT vs T for the C^{oIII}-Re^{IV} complex (7). Data collected under an applied DC field of 0.1 T.

The χT product of the Co^{III}-Re^{IV} compound (7) at 300 K and an applied field of 0.1 T is 1.59 cm^3 K mol^{-1}. This is the expected value for a magnetically isolated Re^{IV} complex with a spin ground state of 3/2 and a g-factor between 1.8 and 1.9, with a low spin (S = 0) Co^{III} ion. The χT product remains near constant down to 50 K where the value drops to 0.38 cm^3 K mol^{-1} at 1.8 K. The drastic decrease in the xT value is attributed to zero field splitting of the Re^{IV} complex and potential weak antiferromagnetic interactions between metal centers. The drop below 1.00 cm3 K mol-1 indicates the existence of these interactions.

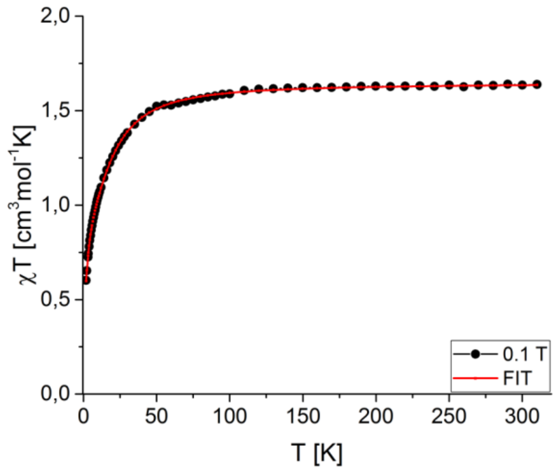

Figure 12 - χT vs T for the C^{oIII}-Re^{IV} complex (8). Data collected under an applied DC field of 0.1 T.

The χT product of the Co^{III}-Re^{IV} compound (8) at 300 K and an applied field of 0.1 T is 1.64 cm^3 K mol^{-1}. This is the expected value for a magnetically isolated Re^{IV} complex with a spin ground state of 3/2 and a g-factor between 1.8 and 1.9, with a low spin (S = 0) Co^{III} ion. The χT product remains near constant down to 50 K where the value drops to 0.60 cm^3 K mol^{-1} at 1.8 K. The drastic decrease in the xT value is attributed to zero field splitting of the Re^{IV} complex and potential weak antiferromagnetic interactions between metal centers. The drop below 1.00 cm3 K mol-1 indicates the existence of these interactions.
